# Bilateral Synchronous Granulomatous Orchitis in a Patient with Erectile Disfunction: Clinical and Pathologic Study of the Case

**DOI:** 10.1155/2013/590608

**Published:** 2013-10-28

**Authors:** M. Rodriguez Peña, D. Moreno

**Affiliations:** Department of Urology, Central Military Hospital, Luis M. Campos 726, C1426BOR Buenos Aires, Argentina

## Abstract

A 50-year-old male patient presented with erectile failure and loss of libido. In the physical examination, there were stone-hard indurations in his bilateral testes. The ultrasonographic study demonstrated multiple hypoechoic areas in the testes and normal epididymis. Since the lesion was presumed as malignancy, bilateral inguinal exploration was performed and intraoperative frozen biopsies were studied and diagnosed as inflammatory process. Nevertheless, we decided to perform left orchiectomy to a deeper histopathologic analysis which revealed granulomatous orchitis, mastocytosis, and severe depletion of Leydig cells at the testicular interstitium. Differential diagnosis between testicular tumor and granulomatous orchitis is very difficult in any examination except by histological findings. Bilateral cases of this pathology are relatively rare, but it is necessary to distinguish them from the testicular tumor before surgical intervention to avoid an unnecessary orchiectomy.

## 1. Introduction

Inflammatory pathology is rare in the testis, and usually, when it is detected, the involvement of the Leydig cells in the process is so infrequent [1]. Most of these inflammatory lesions increase the consistence of the testis and seem to be a testicular tumour [2, 3].

Imagenological analysis such as ultrasonography and colour doppler sonography is not always conclusively in the differential diagnosis between tumoral and non tumoral testicular lesions. Since these lesions are presumed as malignancy, inguinal exploration of the testis is necessary.

## 2. Clinical Case and Results

**Figure 1:**
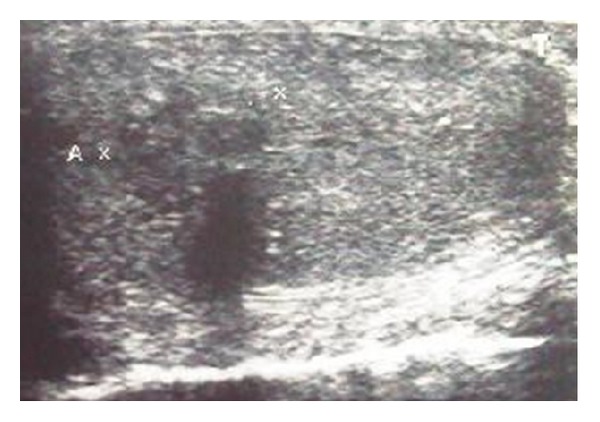
Testicular ultrasonography. Multiple hypoechoic areas in the testes. Both epididymides were normal.

A 50-year-old man presented with erectile failure and loss of libido. There are not antecedents of autoimmune or rheumatoid illness. In the physical examination, there were stone-hard indurations in his bilateral testes. The ultrasonographic study demonstrated multiple hypoechoic areas in the testes and normal epididymis (Figure 1). The values of **α**-fetoprotein and **β**-human chorionic gonadotrophin were normal, but levels of testicular axis hormones were altered (FSH 35.06 UI/mL, LH 19.35 UI/mL, and testosterone 0.40 mg/mL).

**Figure 2:**
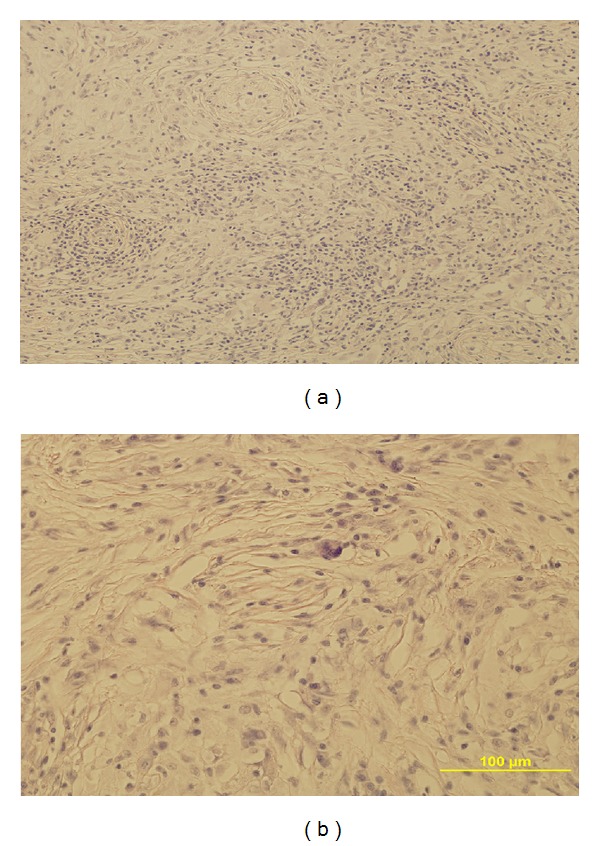
(a) Testis section showing severe stromal fibrosis with the presence of large infiltrates of lymphocytes, macrophages, and eosinophils. Seminiferous tubules are damaged with extended germinal cell loss. H-E 10x. (b) We observed several giant cell clusters and a severe Leydig cell depletion at the interstitium. H-E 40x.

**Figure 3:**
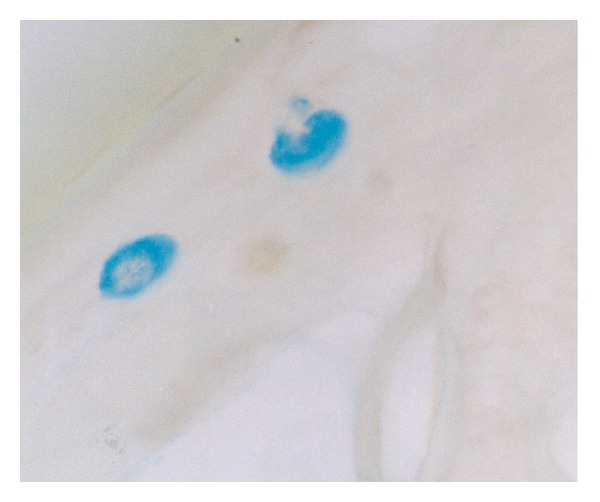
Testicular mast cells are observed near a damaged seminiferous tubule. Alcian Blue technique 100x.

Since the lesion was presumed as malignancy, bilateral inguinal exploration was performed and intraoperative frozen biopsies were studied and diagnosed as inflammatory process. The later histopathology from the biopsy of the left testis revealed histoarchitecture altered with seminiferous tubules hyalinized and atrophic. A severe stromal fibrosis was seen with the presence of large infiltrates of lymphocytes, macrophages, and eosinophils. Also, we observed several giant cell clusters and a severe Leydig cell depletion at the interstitium (Figures 2(a) and 2(b)). By histochemistry using PAS technique, we did not see microorganisms, but using the alcian blue method, we observed a severe mast cell infiltrate mainly around degenerative seminiferous tubules (Figure 3).

The histopathologic diagnosis was bilateral granulomatous orchitis.

We resolved to conserve the right testis in order to preserve the endocrinologic testicular function. Further controls were normal. The patient was under replacement hormonal therapy with testosterone enanthate, and he recovery sexual function and libido.

## 3. Conclusions

The idiopathic granulomatous orchitis is an entity of unknown etiology, clinically or ultrasonographically not distinguishable from testicular tumors. Usually, the differential diagnosis is done by the histological findings.

Bilateral cases of this entity are relatively rare, and, in our knowledge, it is the first report describing this entity as a cause of primary hypogonadism and Leydig cell loss.

Testicular inflammatory processes were described in animal models of experimental autoimmune orchitis (EAO) [4] and in other experimental conditions as testicular torsion [5]. In EAO, granulomas have been observed as clusters of multinucleated cells, mainly spermatids and spermatocytes with degenerative cellular changes [4].

In the human testis, inflammatory cells have been found in some viral conditions as mumps orchitis [6] and in biopsies performed in infertile patients.

Lymphocytes, macrophages, and mast cell infiltrates have been found in association with degenerative seminiferous tubules, and this process is explained as an alteration of the blood testis barrier that permits the passage of antigens from the adluminal tubular compartment to the testicular interstitium [7, 8].

Leydig cell loss is infrequent in the testicular inflammation, and it has not been described in models of EAO. The role of mast cells in normal and pathological testis is not fully understood; however, the number of these cells increases in EAO [9] and in testicular biopsies of patients with infertility [8], suggesting that mast cells might be involved in the testicular damage. It has been demonstrated that mast cell tryptase can increase microvascular permeability and stimulate inflammatory cell migration and cytokine release [10].

We conclude that bilateral granulomatous orchitis is a rare pathology, but it should be considered in the differential diagnosis of diffuse testicular hypo echoic lesions. Histopathological findings suggest that it could be an autoimmune condition, and inflammatory cells, mainly macrophages and mast cells, could play an important role triggering germ cell damage and also being involved in the induction of fibrosis of the wall of seminiferous tubules, as it has been suggested for other testicular pathologies [8].

A correct preoperative diagnosis is necessary to preserve the remainder endocrine function of the testis.
